# A selective aryl hydrocarbon receptor modulator 3,3'-Diindolylmethane inhibits gastric cancer cell growth

**DOI:** 10.1186/1756-9966-31-46

**Published:** 2012-05-16

**Authors:** Xiao-Fei Yin, Jie Chen, Wei Mao, Yu-Hong Wang, Min-Hu Chen

**Affiliations:** 1 Department of Gastroenterology, the First Affiliated Hospital of Sun Yat-Sen University, 58 Zhongshan II Road, Guangzhou, 510080, People’s Republic of China

**Keywords:** Aryl hydrocarbon receptor, 3,3'-Diindolylmethane, Gastric cancer, Cytochrome P4501A1

## Abstract

**Background:**

Aryl hydrocarbon receptor (AhR) is a ligand-activated transcription factor associated with gastric carcinogenesis. 3,3'-Diindolylmethane (DIM) is a relatively non-toxic selective AhR modulator. This study was to detect the effects of DIM on gastric cancer cell growth.

**Methods:**

Gastric cancer cell SGC7901 was treated with DIM at different concentrations (0,10,20,30,40,50 μmol/L) with or without an AhR antagonist, resveratrol. The expression of AhR and Cytochrome P4501A1 (CYP1A1), a classic target gene of AhR pathway, were detected by RT-PCR and Western blot; cell viability was measured by MTT assay, and the changes in cell cycle and apoptosis were analyzed by flow cytometry.

**Results:**

RT-PCR and western-blot showed that with the increase of the concentration of DIM, AhR protein gradually decreased and CYP1A1 expression increased, suggesting that DIM activated the AhR pathway and caused the translocation of AhR from cytoplasm to nucleus. MTT assay indicated that the viability of SGC7901 cells was significantly decreased in a concentration- and time-dependent manner after DIM treatment and this could be partially reversed by resveratrol. Flow cytometry analysis showed that DIM arrested cell cycle in G1 phase and induced cell apoptosis.

**Conclusion:**

Selective aryl hydrocarbon receptor modulator 3,3'-Diindolylmethane inhibits SGC7901 cell proliferation by inducing apoptosis and delaying cell cycle progression. AhR may be a potential therapeutic target for gastric cancer treatment.

## Background

Gastric cancer is one of the most common malignancy. In the economically developping countries, gastric cancer is the second most frequntly diagnosed cancers and the third leading cause of cancer death in males [1], the overall 5-year survival rate is low (15% to 35%) because of the high recurrence rates, nodal metastasis and the short-lived response to chemotherapy [2]. In the present, more and more studies focus on the molecular diagnosis and therapy of gastric cancer [3].

Aryl hydrocarbon receptor (AhR) is a ligand-activated transcription factor. After ligands such as polycyclic aromatic hydrocarbons (PAH) and halogenated hydrocarbons (HAH) bind with AhR in cytoplasm, the ligand-AhR complex is translocated to the nucleus and heterodimerizes with the AhR nuclear translocator (ARNT). The complex binds to the cognate enhancer sequence and subsequently activates downstream gene expression [4].

Traditional studies of AhR function focused on its role in regulating the expression of xenobiotic metabolizing enzymes (XMEs) and mediating the xenobiotics metabolism. Recent studies demonstrated that AhR may involve in many important physiological and pathological processes including individual development, cell differentiation, and carcinogenesis [5]. AhR expression is upregulated in lung [6], mammary gland [7], pancreatic [8] and gastric cancers [9]. Further studies found that AhR played improtant roles in regulating cellular proliferation, apoptosis, cell cycle, migration and invasion [10]. As a protein related to cancer, AhR maybe a promising target for cancer therapy. Our previous work found that an AhR agonist, 2,3,7,8 –tetrachlorodibenzo -para-dioxin (TCDD), inhibited gastric cancer cell growth [9]. But TCDD itself is carcinogenic [11], So to find non-toxic or low-toxic AhR modulators may be a new direction for molecular-targeted therapy in gastric cancer.

Selective AhR receptor modulator 3,3'-Diindolylmethane (DIM) is a class of relatively non-toxic indole derivatives. DIM is an acid-catalyed consendation product of indole-3-carbinol, a consititudent of cruciferous vegetables, and is formed in the stomach [12]. DIM is an anti-cancer agent, it suppresses cancer cell proliferation in mammary [13], colon [14] and pancreatic [15] cancers.

There had been little reports about the effects of DIM on gastric cancer cells growth, the present study was designed to observe the effects of DIM on gastric cancer cells growth and explore the possible mechanisms.

## Methods

### Cell line

Human gastric cancer cell line SGC7901 was obtained from the Cancer Institute of Chinese Academy of Medical Science. SGC7901 Cells were maintained in RPMI-1640 medium (GIBCO, Carlsbad, Calif, USA) supplemented with 10% fetal bovine serum (Hyclone, USA), 1 × 10^5^ U/L of penicillin, and 0.1 g/L of gentamycin. The cellular environment was maintained at 50 mL/L CO2 and 37°C.

### Treatment of cells

DIM was purchased from Enzo Life Science company (Bulter Pike plymouth meeting, PA, USA), resveratrol and dimethyl sulfoxide (DMSO) were purchased from Sigma Chemical Company (Bellefonte, PA, USA). DIM and resveratrol were dissolved in DMSO. After incubating for 24 h, one group of cells was treated with DIM at different concentrations (0, 10, 20, 30, 40, 50 μmol/L) for 24 hours. A second group was treated with DIM (30 μmol/L) plus resveratrol (0, 1, 5, 10, 20 μmol/L) for 6 h. Another group was treated with DIM (30 μmol/L) for different time intervals (0, 1, 6, 24, 48, 72 h), respectively. Control cells received 1 mL/L DMSO only.

### Reverse transcription–polymerase chain reaction (RT-PCR)

After harvesting the cell, total RNA was extracted using the Qiagen RNeasy Mini Kit (Qiagen, Germany) according to the manufacturer’s instructions. cDNA was synthesized with 1 μg total RNA using reverse transcriptase, ReverTraAceTM (Toyobo Co., Osaka, Japan) under the following conditions: 30°C for 10 min, 42°C for 20 min, 99°C for 5 min, and 4°C for 5 min. Polymerase chain reaction (PCR) was performed using 2 μl of complementary DNA and 0.6 U Ex Taq DNA polymerase (Takara, Dalian, China ) in 20 μl reaction system and for 30 cycle with 94°C denaturation for 30 s, 55°C annealing for 30 s and 72°C elongation for 45 s.

The primer sequences were as follows: reverse transcription–polymerase chain reaction (RT–PCR): AhR, 5’- ACT CCA CTT CAG CCA CCA TC -3’ (forward) and 5’- ATG GGA CTC GGC ACA ATA AA -3’ (reverse), the proposed size of PCR product was 204 bp. CYP1A1, 5’- CCA TGT CGG CCA CGG AGT T -3’(forward) and 5’- ACA GTG CCA GGT GCG GGT T -3’ (reverse), the proposedsize of PCR product was 174 bp. Glyceraldehyde-3-phosphate dehydrogenase (GAPDH), 5’- GGG AAA CTG TGG CGT GAT -3’(forward) and 5’- AAA GGT GGA GGA GTG GGT -3’ (reverse), the prospected size of PCR product was 309 bp. PCR products were subsequently electrophoresed on a 1.5% agarose gel, and visualized under a UV transilluminator.

### Western blot analysis

Cells were lysed in buffer containing 20 mmol/L HEPES, 1 mmol/L EGTA, 50 mmol/L β-glycerophosphate, 2 mmol/L sodium orthovanadate, 100 mL/L glycerol, 10 mL/L Triton X-100, 1 mmol/L DTT, and 1 × Protease Inhibitor Cocktail (Roche, Mannheim, Germany). The lysate was centrifuged at 13 000 g and 4°C for 10 min. The supernatant was the total cell lysate. Protein concentration was measured using the BCA protein assay kit (Pierce Chemical Co., Rockford, IL, USA). Thirty micrograms of protein was loaded per lane, separated by 100 g/L SDS-PAGE, and transferred onto equilibrated polyvinylidene difluoride membrane by electroblotting. Membranes were blocked with 5% non-fat milk in 1% TBS-T buffer for 2 h at room temperature. AhR, CYP1A1, and GAPDH were detected for 2 h using antibodies against AhR (SC-5579, Santa Cruz Biotechnology, USA, working dilution 1:150), CYP1A1 (AB1258, Chemicon International, USA, working dilution 1:500), and GAPDH (2118, Cell Signaling Technology, USA, working dilution 1:1000). After secondary antibody incubation (7074,Cell Signaling Technology, USA, working dilution 1:2000) for 2 h, protein bands were detected using ECL system (Pierce Biotechnology, Inc., USA).

### Cell viability assay

The effect of DIM on the proliferation of gastric cancer cells was determined by MTT assay. Briefly, A total of 1 × 10^4^ trypsin-dispersed cells in 0.1 mL culture medium were seeded into each well of a 96-well plate and cultured for 24 hours. Next, cells were treated with DIM as described above. Then, 20 μL of MTT (5 g/L) was added to each well and the incubation was continued for 4 h at 37°C. Finally, the culture medium was removed and 150 μL of DMSO was added to each well. The absorbance was determined with an ELISA reader at 490 nm. The cell viability percentage was calculated as: Viability percentage (%) = (Absorption value of experiment group)/(Absorption value of control group) × 100%.

### Flow cytometric analysis

SGC7901 cells were plated on 60-mm diameter culture plates and treated with DIM at different concentration (10, 20, 30, 40, 50 μmol/L) for 48 h. The control contained 1 mL/L DMSO only. Prior to harvesting, the cells were washed twice with

0.01 mol/L PBS, trypsinized, and pelleted. The cells were then fixed with 70% ice-cold ethanol at 4°C overnight. Finally, the cells were washed twice with PBS and dyed with PI. The DNA content was analyzed with a flow cytometer (Beckman-Coulter, Brea, USA). The cell cycle of SGC7901 cells were analyzed using MULTYCYCLE and winMDI2.9 software (Phoenix, AZ, USA). For cell apoptosis analysis, after incubation for 48 h, cells were stained with annexin V-FITC and PI. Cells with annexin V (−) and PI (−) were deemed viable cells. Cells with annexin V (+) and PI (−) were deemed early apoptotic cells. Cells with both annexin V (+) and PI (+) were deemed late apoptotic cells.

### Statistical analysis

All quantitative data were expressed as mean ± SD and analyzed using a one-way analysis of variance (ANOVA). All statistical analyses were carried out using the SPSS statistical software package (version 11.0, SPSS Inc. Chicago, USA). P < 0.05 was considered statistically significant.

## Results

### Activation of AhR pathway by DIM

**Figure 1:**
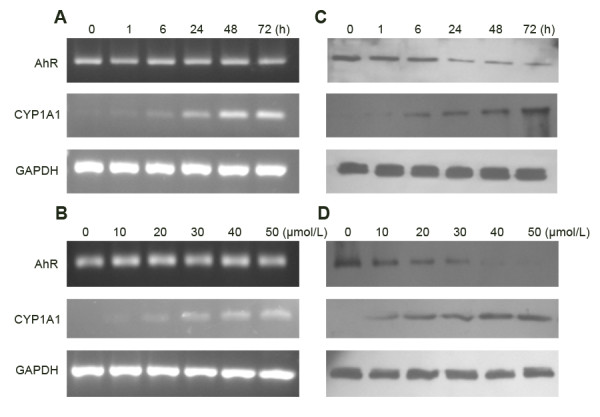
**AhR and CYP1A1 expression in SGC7901 cells after DIM treatment. A** and **B:** RT-PCR; **C** and **D:** Western blotting. Treatment of SGC7901 cells with AhR modulator DIM resulted in a time - (A and C) and concentration -dependent (B and D) induction of CYP1A1 expression. The results shown are representative of three independent experiments.

**Figure 2:**
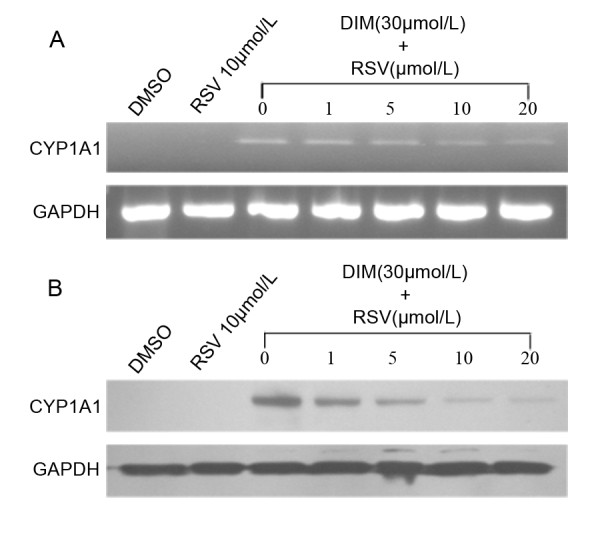
**Inhibition of DIM -induced CYP1A1 mRNA and protein expression by resveratrol. A:** CYP1A1 mRNA was detected by RT-PCR; **B:** CYP1A1 protein was detected by Western blotting. RSV: resveratrol . The results shown are representative of three independent experiments. Treatment of SGC7901 cells with 30 μmol/L DIM caused a remarkable increase in CYP1A1 expression. This DIM-induced CYP1A1 expression was partially reversed by resveratrol in a concentration-dependent manner.

To test whether the AhR signal pathway could be activated by DIM, we treated the gastric cancer cell line SGC7901 with DIM. RT-PCR and Western blot analysis showed that after DIM treatment, AhR protein in the total cell lysates gradually decreased (Figure 1). CYP1A1, a classic target gene of AhR, was utilized as an indicator of AhR signal pathway activation. The baseline level of CYP1A1 expression was not observed in SGC7901 cells, but both CYP1A1 mRNA and protein expression were increased in a dose- and time-dependent manner following DIM treatment (Figure 1). To further confirm the DIM-induced CYP1A1 expression was AhR-dependent, we treated SGC7901 cells with a specific AhR antagonist, resveratrol [16,17]. cells were treated with DIM (30 μmol/L) only or DIM (30 μmol/L) plus different concentrations of resveratrol (0, 1, 5, 10, 20 μmol/L), respectively for 6 h (Figure 2). In concordance with previous results, treatment of SGC7901 cells with 30 μmol/L DIM caused a remarkable increase in CYP1A1 expression. However, this DIM-induced CYP1A1 expression was partially reversed by resveratrol in a dose-dependent manner (Figure 2A and B).

### Effect of DIM on cellur proliferation

**Figure 3:**
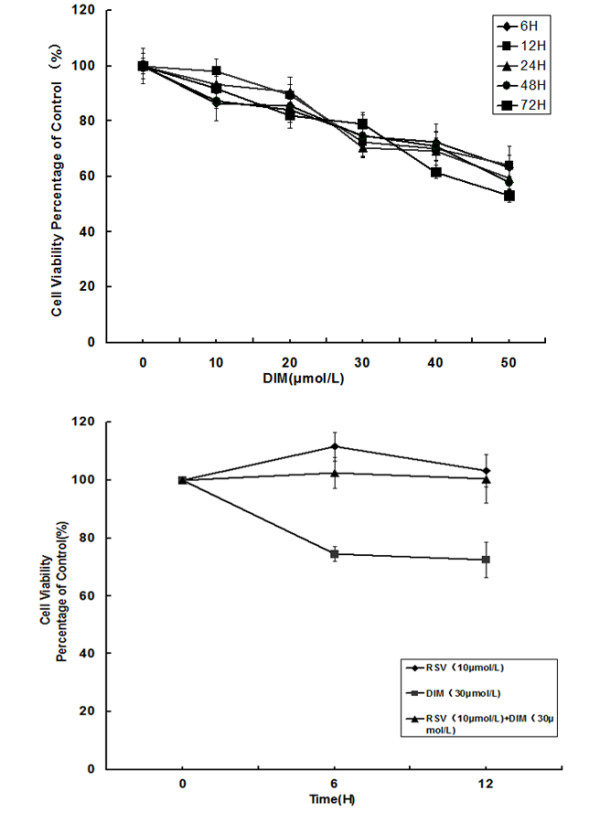
**Viability of SGC7901 cells after DIM treatment was assessed by MTT assay.** Viability of SGC7901 cells was significantly decreased in a concentration- and time-dependent manner after DIM treatment. Resveratrol(10 μmol/L) could partially reverse the inhibition effects of DIM(30 μmol/L) on cellur proliferation.

Proliferation of SGC7901 cells was determined by MTT assay after 6–72 h of treatment with increasing concentrations of DIM (0–50 μmol/L). Results showed that DIM inhibited SGC7901 cellular proliferation in a concentration- and time-dependent manner, Resveratrol (10 μmol/L) could partially reverse the inhibition effects of DIM (30 μmol/L) on cellur proliferation at the time points: 6 h and 12 h (Figure 3), but we did not find the reversal effects at other time points (24 h, 48 h and 72 h, data were not shown).

### Effect of DIM on cell cycle

**Figure 4:**
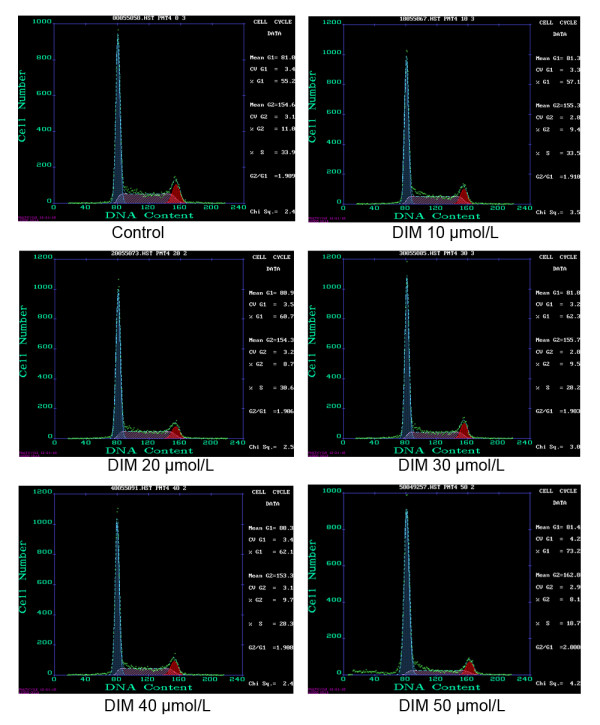
**The effect of DIM on cell cycle of SGC7901 cells.** SGC7901 cells were treated with different concentrations of DIM and subjected to flow cytometric analysis. The percentage of each phase is indicated in each panel. The results shown are representative of three independent experiments.

Flow cytometric analysis revealed that DIM treatment induced changes in cell cycle distribution, with increased accumulation of SGC7901 cells in the G1 phase and compensation for this change by a decrease of cells in the S phase (Figure 4 and Table 1).

**Table 1:** The effect of DIM on cell cycle of SGC7901 cells

DIM concentration (μmol/L)	Percentage of cell cycle (%)		
	G1	G2	S
0	55.90 ± 1.48	10.5 ± 0.95	33.63 ± 0.55
10	57.20 ± 0.36*	9.10 ± 0.3	33.70 ± 0.53
20	61.03 ±1.53*	8.17 ± 0.68	30.77 ± 0.97*
30	61.97 ± 0.32*	9.83 ± 0.32	28.23 ± 0.60*
40	62.77 ± 1.46*	9.13 ± 0.91	28.10 ± 0.56*
50	73.03 ± 4.05*	9.17 ± 1.51	18.07 ± 0.57*

*p < 0.05, vs the control.

### Effect of DIM on cell apoptosis

**Figure 5:**
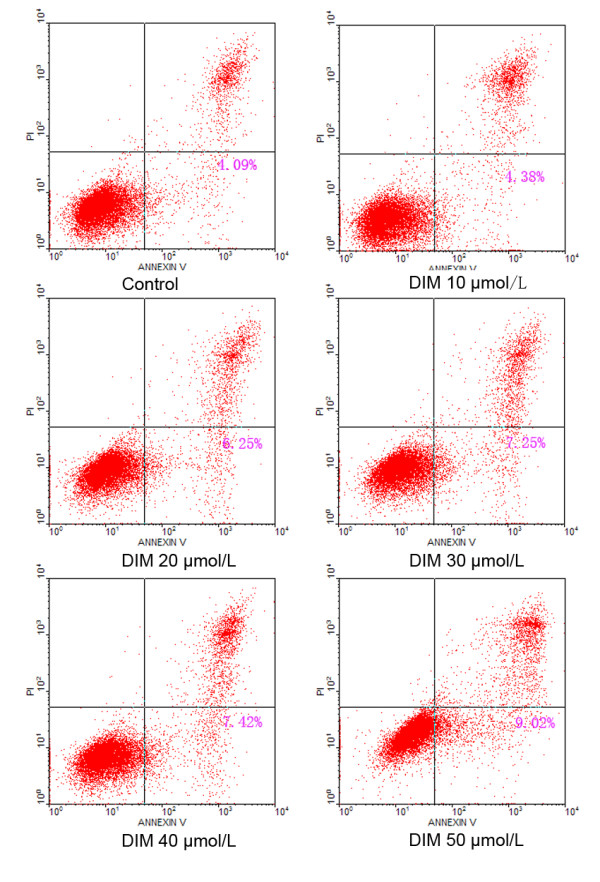
**The effect of DIM on apoptosis of SGC7901 cells.** SGC7901 cells were treated with different concentrations of DIM and subjected to flow cytometric analysis. The results shown are representative of three independent experiments.

48 h after DIM treatment, the changes of cell apoptosis were observed by flow cytometric analysis. Compared to the control group, cell apoptosis was induced at concentrations of 20 to 50 μmol/L, and the apoptosis rate increased in a dose-dependent manner. These results showed that DIM could induce cell apoptosis in SGC7901 cells (Figure 5 and Table 2).

**Table 2:** The effect of DIM on apoptosis of SGC7901 cells

DIM concentration (μmol/L)	Apoptosis rate (%)
0	4.18 ± 0.23
10	4.81 ± 0.42
20	6.07 ± 0.33*
30	7.23 ± 0.78_#_
40	7.39 ± 1.08_#_
50	9.14 ± 0.32_#_

_*_p < 0.05, _#_p < 0.01vs the control.

## Discussion

Our previous work found that the expression of AhR was significantly up-regulated in gastric cancer, and may be involved in the early stage of gastric carcinogenesis, regulation of the AhR pathway may have a potential role in the treatment of gastric cancer. We hypothesized that AhR ligands may be utilized for gastric cancer therapy. Then our futher studies showed that TCDD, a potent AhR agonist, could supresse the growth of gastric cancer cell AGS in a dose- and time-depengent manner via induction of growth arrest at the G1-S phase [9]. But TCDD itself is carcinogenic, it induces a broad spectrum of biological responses, including induction of CYP1A1, disruption of normal hormone signaling pathways, reproductive and developmental defects, immunotoxicity, liver damage, wasting syndrome, and cancer [18], so non-toxic or low-toxic selective AhR modulators maybe served as possible agents for gastric cancer.

From the studies in breast cancer, Safe found two classes of selective AhR modulators: Alternate-substituted (1,3,6,8- and 2,4,6,8-) alkyl polychlorinated dibenzofurans (PCDFs) and substituted diindolylmethanes (DIMs), compared to TCDD, these compounds are relatively non-toxic and inhibit ER-positive and ER-negative mammary tumor growth, but do not induce AhR-mediated toxic responses induced by TCDD [19].

DIM represents a new class of relatively non-toxic antitumorigenic AhR modulators which are of phytochemical origin. Compared to TCDD, DIM is a weak agonist of AhR for induction of CYP1A1 gene expression [20] and activities [21], and it shows abilities to compete for binding of TCDD to the AhR [22].

To test wether the AhR signal pathway could be activited by DIM in gastric cancer cells, we treated gastric cancer cell line SGC7901 with DIM. Results showed that AhR protein in the total cell lysates gradually decreased (Figure 4C and D), Similar phenomena have been reported by our labs and several other groups [9,23], the down-regulation of AhR following ligand binding is regarded as an imprtant step of AhR signal pathway [23]. CYP1A1, a classic target gene of AhR, was chosen as an indicator of AhR signal pathway activation. Baseline levels of CYP1A1 expression were not observed in SGC7901 cells in the present study. However, expression of CYP1A1 was significantly increased in a concentration- and time-dependent manner after DIM treatment, indicating the activation of AhR. To confirm the activation of the AhR signal pathway by DIM, we treated SGC7901 cells with a specific AhR antagonist, resveratrol. Our results showed that DIM -induced CYP1A1 expression was partially reversed by resveratrol in a concentration-dependent manner. The incomplete reversal of CYP1A1 expression by resveratrol may be due to the fact that AhR is not the only regulator of CYP1A1 transcription [24]. Taken together, these results suggest that DIM could activate the AhR signal pathway in gastric cancer cells.

MTT assay demonstrated that the viability of SGC7901 cells was significantly decreased in a concentration- and time-dependent manner after DIM treatment. To further clarify wether this effects was AhR- dependent, we treated SGC7901 cells with DIM and resveratrol, we found that the inhibition effects of DIM on SGC7901 cells growth was partially but not completely reserved by reservatrol, suggesting that DIM inhibits gastric cancer cell growth partially via AhR pathway. This result is in accordance with previous studies: Hong,C found that DIM inhibited growth of both Ah-responsive and Ah-non-responsive breast cancer cells. some of the anti-carcinogenic activities of DIM are AhR –independent [25]. Interestingly, the reversal effect on cell proliferation was observed after cells were treated with DIM plus reservatrol for 6 h or 12 h, but not at longer time points (24 h, 48 h and 72 h), this maybe related to the time-effectiveness of reservatrol.

There have been some studies about the anti-cancer machanisms of DIM. Choi HJ showed that DIM induced G1 and G2/M phase cell cycle arrest in HT-29 human colon cancer cells [26]. Vivar OI and Hong C found DIM induced a G(1) arrest in human prostate cancer cells [27] and human breast cancer cells [28]. On the other hand, some articles reported that DIM may promote apoptosis in cancer cells by survivin , uPA and uPAR or NF-kappaB sinaling [29-33].

To further explore the specific mechanisms of gastric cancer cell growth inhibition by DIM, we treated SGC7901 cells with DIM, then tested the changes of cell cycle and cell apoptosis by flow cytometric analysis. The results showed that with the increase of DIM concentration, cells in G1 phase gradually increased, cells in S phase decreased, but cells in G2 phase remained unchanged, indicating that DIM could arrest cell cycle in G1 phase. Different from TCDD, DIM also induced cell apoptosis, suggesting that DIM could suppress gastric cancer cell proliferation through inducing apoptosis and arresting cell cycle, However, the mechanisms responsible for the effects of DIM on gastric cancer cell cycle and apoptosis are still needed to be further studied.

## Conclusions

In surmary, this report showed that non-toxic selective AhR modulator DIM inhibited the proliferation of human gastric cancer cell line SGC7901 in vitro by inducing cell apoptosis and arresting cell cycle at G1 phase. Our findings suggested that AhR might be a promising target for gastric cancer treatment.

## Abbreviations

AhR: Aryl hydrocarbon receptor; GAPDH: Glyceraldehyde 3-phosphate dehydrogenase; RT-PCR: Reverse Transcription -Polymerase Chain Reaction; MTT: Methyl thiazolyl tetrazolium; DMSO: Dimethyl sulfoxide; FBS: Fetal bovine serum; TCDD: 2,3,7,8-tetrachlorodibenzo-para-dioxin; TBST: Tris-buffered saline containing 0.05% Tween 20.

## Competing interests

The authors declare that they have no competing interests.

## Authors’ contributions

Yin XF and Chen J contributed equally to this work; Yin XF and Chen J performed research and wrote the paper; Mao W organized the figures and analyzed data; Wang YH assisted with cell culture. Chen MH designed research and supervised the writing and organization process. All authors read and approved the final manuscript.
